# Longitudinal increases in mitochondrial DNA levels in blood cells are associated with survival in critically ill patients

**DOI:** 10.1186/cc6096

**Published:** 2007-08-15

**Authors:** Hélène CF Côté, Andrew G Day, Daren K Heyland

**Affiliations:** 1 Department of Pathology and Laboratory Medicine, University of British Columbia, Vancouver, Canada V6T 2B5; 2 Clinical Research Centre, Kingston General Hospital, Kingston, Canada K7L 2V7; 3 Department of Medicine, Queen's University and Critical Care Program, Kingston General Hospital, Kingston, Canada K7L 2V7

## Abstract

**Background:**

Mitochondrial dysfunction may be causally related to the pathogenesis of organ failure in critically ill patients. Decreased mitochondrial DNA (mtDNA) levels have been associated with mitochondrial dysfunction and were investigated here in relation to short-term (31-day) survival.

**Methods:**

This was a prospective longitudinal cohort study of 28 mechanically ventilated critically ill adults admitted to a single center tertiary care intensive care unit (ICU) with hypotension secondary to cardiogenic (*N *= 13), septic (*N *= 14) or hypovolemic (*N *= 1) disease processes. Clinical data and blood were collected at baseline and patients were followed until they expired or left the ICU. Blood was collected every Monday, Wednesday and Friday, and the buffycoat relative mtDNA/nuclear DNA (nDNA) ratio was determined. An archived pool of healthy controls was also studied.

**Results:**

At baseline, the patients' mtDNA/nDNA ratio was 30% lower than a pool of 24 healthy controls (0.76 versus 1.09) and was not different between short-term survivors and non-survivors (0.74 ± 0.05 (*N *= 16) versus 0.79 ± 0.06 (*N *= 12), *p *= 0.49). By day 4, the percent mtDNA/nDNA change from baseline in survivors was significantly different from that in non-survivors (+29.5% versus -5.7%, *p *= 0.03). It also tended to be higher in survivors at last measurement (+38.4% versus +7.1%, *p *= 0.06). There was a weak correlation between within patient mtDNA/nDNA and platelet count (r = 0.20, *p *= 0.03) but not with Sequential Organ Failure Assessment (SOFA) scores (r = 0.12, *p *= 0.23). The mtDNA associations remained after adjustment for platelet.

**Conclusion:**

Blood mtDNA levels appeared initially low, increased over time in patients who ultimately survived, and remained low in those who did not. This is consistent with mitochondrial recovery being associated with survival and warrants further investigation as a marker of mitochondrial alterations and outcome in critical illness.

## Introduction

It is well known that oxygen consumption appears reduced in critically ill patients [1]. The primary oxygen consumer in human cells is the mitochondrial respiratory chain, which is responsible for 90% of oxygen consumption under normal conditions, and accounts for most of the cellular ATP production. The facts that optimized tissue oxygenation does not prevent organ failure and death [2] and that mitochondrial damage occurs in the absence of hypoxia [3,4] indicate that impaired oxygen utilization by the mitochondria and not only oxygen availability is at play in critical illness.

This bioenergetics failure has been hypothesized as part of the mechanism underlying multiple organ failure and death [5,6] and is supported by several lines of evidence. For example, several animal models of sepsis have demonstrated inhibition of mitochondrial function [7] as well as depletion of the number of heart [8] and liver [9] mitochondria that were not due to cell death. In a rat model of sepsis, mitochondrial DNA (mtDNA) damage and depletion, accompanied by decreased mtDNA transcription, preceded bioenergetics failure while restoration of mtDNA integrity appeared linked to mitochondrial biogenesis [10]. In human volunteers, systemic inflammation following *in vivo *endotoxin administration was associated with widespread transcriptional down-regulation of the mitochondrial energy production machinery [11]. In critically ill patients, strict glucose control with insulin has been associated with reduced mortality [12] and, interestingly, was also shown to prevent hepatic mitochondrial ultrastructural damage [13]. In septic patients, skeletal muscle ATP concentrations and mitochondrial complex I activity were both significantly reduced in individuals who subsequently died compared to septic patients who survived and controls [5]. Finally, another group also found decreased muscle mitochondria content in critically ill patients with sepsis-induced multiple organ failure [14].

In other settings such as HIV antiretroviral therapy, clinically symptomatic mitochondrial dysfunction has been associated with mtDNA depletion [15]. Each tissue contains more or less mitochondria depending on its energy requirement, translating into several hundred to several thousand copies of mtDNA per cell. In sepsis, it has been suggested that excessive oxidative stress in the mitochondria may effect changes in mtDNA quantity [16]. It could also decrease mtDNA quality by causing mutations or deletions. Of note, damaged mitochondria can still replicate, even in the absence of cellular division, and elimination of mitochondria (also termed mitoptosis) can occur, presumably in response to damage at the mitochondrial level, while the cells remain viable [17,18]. Hypothetically, if organ failure was driven by mitoptosis, then a concurrent decrease in mtDNA could be expected while the cells and tissues remain apparently alive yet dysfunctional, up to a point of no return.

The aim of this study was to describe blood cell mtDNA levels in critically ill patients and evaluate their association with intensive care unit (ICU) survival and admission diagnosis. We hypothesized that persistent low blood mtDNA levels are associated with mortality.

## Materials and methods

### Study population

This study is a sub-study of a single center, open-label, phase I, prospective, optimal dose-finding clinical trial of glutamine and antioxidants conducted at the Kingston General Hospital, Kingston, Canada, the details of which were recently published [19]. Consecutive eligible adult patients admitted to ICU within the last 24h, requiring mechanical ventilation, with clinical evidence of hypotension, and expected to stay more than 48h were enrolled. We defined clinical evidence of hypotension as the need for vasopressor agents (norepinephrine, epinephrine, neosynephrine, vasopressin, or ≥5 mg/kg/minute of dopamine) for more than 1h or a systolic blood pressure ≤90 mmHg or mean arterial pressure <70 mmHg for more than 1h despite adequate fluid challenge. Patients were ineligible if they had no gastrointestinal tract access, severe head trauma, cirrhosis, were severely underweight (<50 kg) or pregnant, or if they were already enrolled in another ICU interventional study. Upon enrolment, age, sex, co-morbidities, admission diagnosis, APACHE II [20], and Sequential Organ Failure Assessment (SOFA) [21] scores were recorded. Study participants were part of four groups (*N *= 7 each) who were all given various doses of antioxidant in the form of glutamine dipeptides (Dipeptiven^®^) and selenium (MicroSel^®^) (see [19] for details). The patients were followed closely, and organ dysfunction was calculated daily using SOFA score. Written informed consent was obtained from next of kin to enable participation of eligible patients. This study protocol was approved by the Research Ethics Board at Queen's University.

### Blood sample collection

Venous blood was collected in EDTA at study entry and every Monday, Wednesday, and Friday over the following 28 days, until death or discharge from the ICU. The tubes were spun at 2,500 g for 10 minutes to separate plasma and buffycoat cell pellet. The latter was stored frozen at -70°C until used.

### Assays

Total DNA was extracted from 0.1 ml of buffycoat using a QiaAMP DNA kit (Qiagen Mississauga, Ontario, Canada) from a total of 159 samples collected from 28 individuals. The relative mtDNA/nuclear DNA (nDNA) ratios were determined by real-time PCR with fluorescent probes, as described elsewhere [12,22]. All quantifications were performed on a LightCycler 1.2 (Roche, Laval, Quebec, Canada). The mtDNA content of the cells is expressed as a relative mtDNA/nDNA ratio: the nuclear DNA copy number per cell being considered constant, alteration of the ratio can be attributed to changes in mtDNA content. No control samples from healthy patients were collected as part of this study. However, for the sake of comparison, the mtDNA/nDNA ratio of a DNA pool containing buffycoat DNA from 24 healthy males (average age 39 years old) and used as internal control for the assays showed a mean ± standard deviation relative mtDNA/nDNA ratio of 1.09 ± 0.17. The mtDNA/nDNA ratios or platelet count of the 24 individual blood samples used to generate the pool was not available.

Of note, platelets contain an average of four copies of mtDNA per platelet, but no nDNA [23]. As such, their number and mtDNA content could influence the mtDNA/nDNA ratio in blood [24]. In addition, although there have been conflicting reports about changes in muscle mtDNA content over time [25,26], there have been no reports about changes in blood mtDNA content with age in adults.

### Statistical analyses

Baseline, early (4 ± 1 days), and late (last measurement recorded) values of the mtDNA/nDNA ratio are described in this population. Spearman's partial correlation (controlling for subject) was used to measure within subject correlation between mtDNA and platelet count, as well as mtDNA and SOFA scores. Spearman's correlation was used for baseline mtDNA versus baseline APACHE score correlation. All comparisons between 31-day survivors and non-survivors used the two-sample *t*-test, except for ICU duration and number of samples collected, which used the Wilcoxon-Mann-Whitney non-parametric test due to their strong positive skew. Analysis of covariance was used to repeat the comparison of the mtDNA measurements between survivors and non-survivors after adjusting for platelet counts, white blood cell count, and antioxidant dosing group. In addition, a linear mixed model with random patient intercepts and slopes was used to compare the average slope of the longitudinal mtDNA/nDNA ratio over the first 14 days by survival status. This model was estimated by restricted maximum likelihood as implemented by the MIXED procedure of SAS version 8.2 [27].

## Results

There were 28 subjects enrolled in the study, including 8 females and 20 males, with an average age of 67 years old. Of these, 13 patients (46%) had cardiogenic shock while 14 (50%) were diagnosed with septic shock and 1 (4%) with hypovolemic shock (Table 1). A total of 159 distinct samples were collected longitudinally every two to three days while the patients were in ICU and used for these analyses, for an overall median [interquartile range] of 5.0 [4.0 to 7.5] samples per patient. Of the 28 subjects in the study, 12 patients died within 31 days of ICU admission. Of those who died, 8 had septic and 4 had cardiogenic shock. Over the study period, both survivors and non-survivors had a similar number of samples (5.5 [4.0 to 7.0] versus 4.5 [3.0 to 8.0] (*p *= 0.45)) collected over a similar number of days in ICU (11.5 [7.6 to 17.8] versus12.9 [5.7 to 26.3] (*p *= 0.51)).

**Table 1:** Study subject characteristics

N	28
Age, mean (range)	67 (34–81)
Female, N (percent)	8 (29)
APACHE II score, mean (range)	22.4 (15–37)
Etiology of shock, N (percent)	
Cardiogenic	13 (46)
Septic	14 (50)
Hypovolemic	1 (4)
ICU days, median [IQR]	11.5 [6.3–20.3]
Short-term mortality, N (percent)	12 (43)

ICU, intensive care unit; IQR, interquartile range.

At baseline, the mean baseline relative mtDNA/nDNA ratio for all 28 critically ill patients (0.76) was approximately 30% lower than the ratio measured for the historical pool of control buffycoat of 24 healthy subjects (1.09). There was no difference between the baseline mtDNA/nDNA ratio of survivors and non-survivors (*p *= 0.49; Table 2). At baseline, there was also no correlation between mtDNA and APACHE scores (r = 0.27, *p *= 0.16). The percent change in the mtDNA/nDNA ratio from baseline to day 4 (± 1 day) was significantly greater in survivors (+29.5% versus -5.7%, *p *= 0.03) compared to non-survivors. This was maintained to the last measurement available, where the percent change from baseline also tended to be greater for survivors (+38.4% versus +7.1%, *p *= 0.06).

**Table 2:** Blood mitochondrial DNA/nuclear DNA ratio in short-term survivors versus non-survivors

	No adjustment			Adjusted for platelet count		
		
mtDNA/nDNA ratio	Survivors (*N *= 16)	Non-survivors (*N *= 12)	*P *value	Survivors (*N *= 16)	Non-survivors (*N *= 12)	*P *value
At baseline	0.74 ± 0.05	0.79 ± 0.06	0.49	0.74 ± 0.05	0.79 ± 0.06	0.49
At day 4 (± 1)	0.94 ± 0.08	0.73 ± 0.09	0.10	0.95 ± 0.08	0.72 ± 0.10	0.10
At last measurement	0.98 ± 0.06	0.79 ± 0.07	0.04	0.97 ± 0.06	0.79 ± 0.07	0.05
Change at day 4 (± 1)	+29.5 ± 10.1	-5.7 ± 11.6	0.03	+30.3 ± 10.3	-6.6 ± 11.9	0.03
Change at last measurement	+38.4 ± 10.5	+7.1 ± 12.1	0.06	+36.5 ± 10.4	+9.7 ± 12.0	0.11

Results presented as mean ± standard error. mtDNA, mitochondrial DNA; nDNA, nuclear DNA.

Within patients, a weak but significant partial correlation was observed between blood mtDNA and platelet count (r = 0.20, *p *= 0.03) but not between mtDNA and daily SOFA score (r = -0.12, *p *= 0.23). Given the weak relationship observed between platelet count and mtDNA levels, the influence of the latter on the mtDNA/nDNA ratio was investigated. As seen in Table 2, the associations between mtDNA and survival were largely unaffected after adjusting for platelets. Table 3 further demonstrates that the changes in mtDNA were not driven by changes in platelets. Similar estimates were maintained after adjustment for platelets as well as white blood cell count and antioxidant dosage group, although the statistical significance was lost. After adjustment for platelet and white blood cells, the mean ± standard error change in mtDNA at 4 ± 1 days was +27.9 ± 10.5 for survivors versus -3.5 ± 12.3 for non-survivors (*p *= 0.07), and if the antioxidant group was added, this became +24.4 ± 10.4 versus +1.1 ± 12.2 (*p *= 0.18).

**Table 3:** Blood platelet count in short-term survivors versus non-survivors

Platelet count	Survivors (*N *= 16)	Non-survivors (*N *= 12)	Difference (survivors – non-survivors)	*P *value
At baseline	157 ± 21	176 ± 25	-19 ± 33	0.57
At day 4 (± 1)	139 ± 22	186 ± 26	-48 ± 34	0.18
At last measurement	262 ± 35	239 ± 40	23 ± 53	0.67
Change at day 4 (± 1)	-6 ± 10	+10 ± 12	-16 ± 15	0.32
Change at last measurement	+91 ± 25	52 ± 29	+39 ± 38	0.31

Results presented as mean ± standard error.

**Figure 1:**
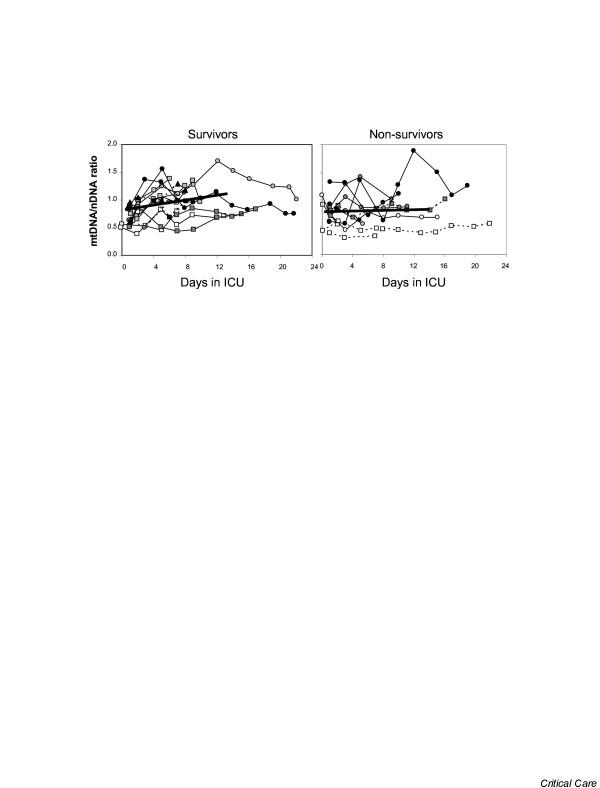
Longitudinal relative blood mitochondrial DNA (mtDNA)/nuclear DNA (nDNA) ratio. Of the 28 critically ill subjects, survivors are presented in **(a) **and non-survivors in **(b)**. Patients admitted with septic shock are represented by circles, those with cardiogenic shock by squares, and the hypovolemic patient by a triangle. Patients belonging to the four antioxidant treatment groups are distinguished by the color of their symbol (white, light grey, dark grey and black). Males are represented by a solid line and females by a dashed line. The thick lines represent the linear modeling of the mean mtDNA/nDNA slopes for the short-term survivors (*N *= 16, solid line) and the non-survivors (*N *= 12, dashed line) over the first 14 days after enrollment. Though the entire duration of data collected is shown on the graph, only data collected up to the first 14 days are used in the linear model shown here. ICU, intensive care unit.

**Figure 2:**
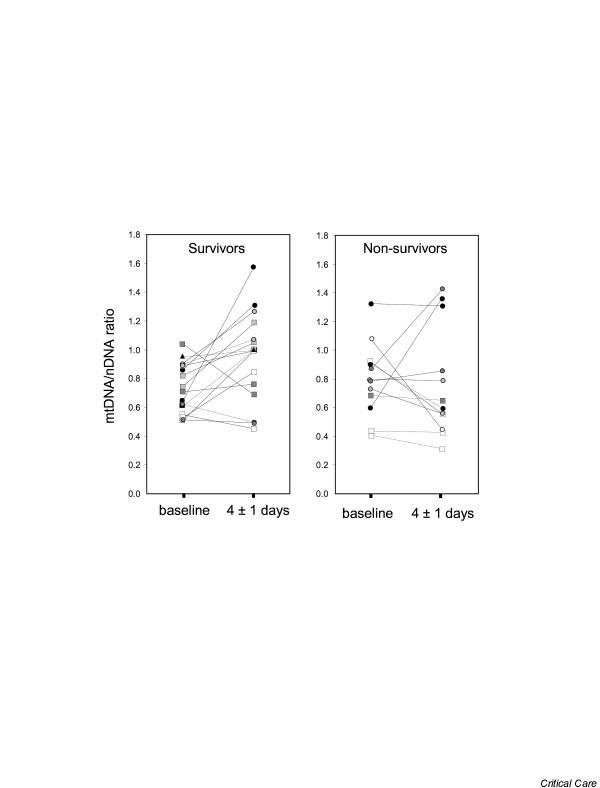
Change in mitochondrial DNA (mtDNA)/nuclear DNA (nDNA) ratio between baseline and day 4 (± 1) in the intensive care unit. Of the 28 critically ill subjects, survivors are presented in **(a) **and non-survivors in **(b)**. Patients admitted with septic shock are represented by circles, those with cardiogenic shock by squares, and the hypovolemic patient by a triangle. Patients belonging to the four antioxidant treatment groups are distinguished by the color of their symbol (white, light grey, dark grey and black).

From the linear mixed model, we estimated that the average slope (estimate ± standard error) of the longitudinal change in mtDNA/nDNA over the first 14 days was statistically significant for survivors (0.024 ± 0.010, *p *= 0.02) but nearly flat for non-survivors (0.002 ± 0.013, *p *= 0.85). However, the difference between the two slopes, as estimated by the interaction between time and survival status, was not statistically significant (0.022 ± 0.016, *p *= 0.18; Figure 1). We repeated the modeling exercise using data from the first 7 and 10 days and our findings were consistent with the exception that the *p *values for the differences between the two slopes were *p *= 0.11 for the 7 day model and *p *= 0.10 for the 10 day model. Similar estimates were also maintained after adjustment for platelet count and antioxidant dosage group (not shown). Figure 2 provides a closer look at the change in buffycoat mtDNA levels between the first sample collected (baseline) and that collected at 4 ± 1 days in the ICU.

## Discussion

On the first day of enrolment, mechanically ventilated critically ill patients with clinical evidence of hypotension had a relative buffycoat mtDNA/nDNA ratio that appeared lower than that of a pool of healthy historical controls, suggestive of some mitochondrial alteration. Of note, the historical control pool is presented for general rather than statistical comparison as it consisted of generally younger individuals whose individual blood mtDNA/nDNA ratio or platelet counts were not known. No significant difference was observed in the baseline blood mtDNA level of those who would survive as opposed to those who would die within the short term. However, those who survived were more likely to experience an increase in blood mtDNA content over time than those who died, suggesting that recovery of mtDNA content is associated with a better outcome.

Blood buffycoats contain platelets, which in turn contain a small amount of mtDNA. It is not unexpected, therefore, to observe some relationship between platelet count (which is often affected in critical illness) and mtDNA levels. However, the associations observed here were all maintained after adjusting for platelet count. Trends were also maintained after adjusting for antioxidant dosage and white blood cell count.

The mechanism by which mtDNA is depleted in this patient population and the chronology of the molecular events involved is unclear. More than one scenario can be hypothesized. On the one hand, the apparent decrease in mtDNA content at baseline could reflect an early event associated with shock and causing mtDNA damage. A potential pathway for shock-related mitochondrial damage would be through the toll-like receptor 4 (TLR4) [28]. TLR4 has been shown to mediate mtDNA damage through increased oxidative stress and inducible nitric oxide synthase expression in a mouse model of bacterial sepsis [29]. Changes in TLR4 expression and/or signaling have also been implicated in hemorrhagic shock in animal models [30-32]. As stressful oxidative conditions to the cell and its mitochondria are induced, this may trigger the elimination of mitochondria from cells, much as observed *in vitro *[18] and more recently *in vivo *[9]. In the former study, when cultured cells were treated for two to three days with inhibitors of bioenergetic functions, such as uncouplers of the mitochondrial respiratory chain, a large percentage (50% to 70%) of cells died but those that survived showed no sign of apoptosis and had very low mtDNA content. Cell death was not caused by lack of energy but the authors suggested hyperproduction of reactive oxygen species as the probable reason for cell death [18]. They hypothesized that cells eliminating mitochondria would show a selective advantage by lowering the content of pro-apoptotic mitochondrial proteins and eliminating the major source of reactive oxygen species. Similarly, in a mouse model, increased lysosomal clearance of damaged liver mitochondria was inferred during the subacute phase of sepsis [9]. The authors suggested a dynamic turnover and replacement of damaged mitochondria over time. Drawing a parallel with patients in shock, depletion of mitochondria (and mtDNA) may be the result of extreme oxidative stress and the damage it causes. Failure to recover mitochondria once the insult is withdrawn would be detrimental to the patient outcome. Antioxidants, through reduction of oxidative stress damage, might offer cellular protection. This would be in agreement with antioxidants having a protective effect on the mitochondria, as has been suggested [16,19] and may partially explain the apparent therapeutic benefit to antioxidant supplementation in critical illness [33,34].

On the other hand, mtDNA depletion may be a relatively late event in response to cell death signaling. Xue and colleagues [35] showed that eukaryotic cells can eliminate their mitochondria in a highly specific manner, without affecting other organelles. Again, if a parallel is made with the condition of shock, severe and persistent mitochondria depletion may be a marker of an irreversible path toward death, possibly by multi-organ dysfunction syndrome. However, in this case, one may have expected stable mtDNA levels in survivors and declining ones in non-survivors, which is not the observation made here.

If fewer mitochondria per cell is at least in part responsible for the apparent mitochondrial dysfunction observed in sepsis, this depletion may also contribute to diminished overall mitochondrial respiratory chain activity and impaired oxygen use or hypoxia, as seen in association with shock. Consistent with this concept, mitochondria-depleted eukaryotic cells have been shown to survive longer in hypoxic conditions [35]. Other mechanisms, such as reduced pyruvate delivery to the mitochondrial tricarboxylic acid cycle or inhibition of mitochondrial enzymes, have also been suggested [36].

Although much more research is required to elucidate the mechanism of mtDNA depletion in shock, this exploratory study would be in agreement with the general hypothesis brought forth by Brealey and Singer [6], namely that the cell might undergo some kind of energy shutdown in an attempt to get through an acute phase. This hypothesis is further supported by the recent observation that during acute systemic inflammation, blood leukocytes undergo widespread transcriptional downregulation of mitochondrial genes involved in energy production [11]. Our results would suggest that cellular depletion of the mitochondria organelles may contribute to the apparent mitochondrial dysfunction noted in shock and multi-organ dysfunction syndrome [5]. The recovery of cellular mtDNA associated with a better survival outcome as observed here may reflect recovery of mitochondria biogenesis and an improvement in overall condition. Whether the mitochondrial recovery seen here in association with survival is driving the improvement in the patient's health status or whether it is a consequence of such improvement cannot be ascertained by our study. Nevertheless, mtDNA levels and their change over time may provide a novel and relatively simple marker of mitochondrial function.

Given its small sample size, this study was exploratory in nature and not designed to allow adjustment for all potential confounding factors of blood mtDNA levels. In addition, *p *values were not adjusted for multiple comparisons. Larger controlled studies of isolated blood cell populations will be needed to confirm the relationship between mtDNA levels and critical illness outcome observed here.

## Conclusion

In critically ill patients requiring ventilation and with clinical evidence of hypotension, we observed low blood mtDNA levels at baseline. Patients who survived at least the following 31 days showed an increase in mtDNA levels while those in patients who did not survive remained low. These results are consistent with the notion that some mitochondrial 'shutdown' may also take place in humans during shock, providing corroborative evidence to observations obtained from animal models. This should spark interest in mtDNA quantification not only as a tool toward a better understanding of the pathogenesis of multiple organ dysfunction syndrome but also as a potential marker of mitochondrial dysfunction in critical illness.

## Key messages

• In patients admitted to ICU in shock, an increase in blood mitochondrial DNA levels over time is associated with short-term survival.

• mtDNA quantification could be explored as a marker of mitochondrial dysfunction in critical illness.

## Abbreviations

APACHE II = Acute Physiologic and Chronic Health Evaluation II score; ICU = intensive care unit; mtDNA = mitochondrial DNA; nDNA = nuclear DNA; SOFA = Sequential Organ Failure Assessment; TLR4 = toll-like receptor 4.

## Competing interests

A patent application has been filed by the University of British Columbia regarding the use of mtDNA quantification I sepsis. HC is an inventor on this patent application, filed in January 2005. HC is employed by University of British Columbia as an Assistant Professor.

## Authors' contributions

All authors participated in the interpretation of the results. DH collected the study samples and the clinical data. HC collected the mtDNA data and wrote the manuscript. AD carried out the statistical analyses and prepared the figures. All authors participated in revising the manuscript.
